# Reduced Functional Reserve in Patients with Age-Related White Matter Changes: A Preliminary fMRI Study of Working Memory

**DOI:** 10.1371/journal.pone.0103359

**Published:** 2014-08-13

**Authors:** Martin Griebe, Michael Amann, Jochen G. Hirsch, Lutz Achtnichts, Michael G. Hennerici, Achim Gass, Kristina Szabo

**Affiliations:** 1 Department of Neurology, MR Research Neurology, UniversitätsMedizin Mannheim, University of Heidelberg, Mannheim, Germany; 2 Department of Neurology, University Hospital Basel, Basel, Switzerland; 3 Division of Diagnostic and Interventional Neuroradiology, Department of Radiology, Basel, Switzerland; 4 Fraunhofer MEVIS, Institute for Medical Image Computing, Bremen, Germany; Institute of Automation, Chinese Academy of Sciences, China

## Abstract

Subcortical age-related white matter changes (ARWMC) are a frequent finding in healthy elderly people suggested to cause secondary tissue changes and possibly affecting cognitive processes. We aimed to determine the influence of the extent of ARWMC load on attention and working memory processes in healthy elderly individuals. Fourteen healthy elderly subjects (MMSE >26; age 55–80 years) performed three fMRI tasks with increasing difficulty assessing alertness, attention (0-back), and working memory (2-back). We compared activation patterns in those with only minimal ARWMC (Fazekas 0–1) to those with moderate to severe ARWMC (Fazekas 2–3). During the fMRI experiments, the study population showed activation in brain areas typically involved in attention and working memory with a recruitment of cortical areas with increasing task difficulty. Subjects with higher lesion load showed a higher activation at all task levels with only sparse increase of signal with increasing complexity. In the lower lesion load group, rising task difficulty lead to a significant and widely distributed increase of activation. Although the number of patients included in the study is small, these findings suggest that even clinically silent ARWMC may affect cognitive processing and lead to compensatory activation during cognitive tasks. This can be interpreted as a reduction of functional reserve and may pose a risk for cognitive decline in these patients.

## Introduction

As cerebral white matter lesions are highly prevalent in healthy elderly individuals – having been reported in 27%–92% of this population [1], [2] – these findings have also been termed age-related white matter changes (ARWMC). Their extent seemingly corresponds to a continuum from normal functioning to clinically overt neurological syndromes in subcortical vascular encephalopathy (SVE) with apraxia of locomotion, gait disturbance, working memory deficits and executive dysfunction [3]. Using diffusion tensor imaging (DTI) and planimetry of the corpus callosum we have recently demonstrated a loss of tissue integrity and atrophy of the corpus callosum secondary to spatially remote – and per se clinically silent – lesions in the peri- and paraventricular white matter in ARWMC in healthy elderly individuals [4]. In a study of cognitive correlates of subclinical structural brain disease in elderly healthy control subjects, even modest volumes of ARWMC were found to be functionally associated with a decline in cognitive performance [5]; in particular, ARWMC have been suggested to be an important cause of age-related attentional and executive dysfunction in the elderly [6]. The functional processes underlying the transition to SVE, with affection of cognitive skills - especially attention, working memory, and cognitive processing speed are poorly understood. Substantial fMRI research into the pathophysiology of aging has focused on task related brain activity changes in the elderly [7], [8]. An intriguing finding is the recruitment of larger brain areas interpreted as compensation – either with or without clinical correlation [9], [10].

The aim of our study was to determine the influence of ARWMC load on attention and working memory task performance with increasing complexity in healthy elderly individuals. We used a three task paradigm with increasing task difficulty (alertness<attention<working memory) and compared subjects with no or mild ARWMC to those with moderate to severe ARWMC. We hypothesized that increasing difficulty of presented tasks might be compensated in patients with progressive ARWMC by extended recruitment of normally silent brain areas.

## Methods

### Ethics Statement

The local ethics committee (Medical Ethics Committee 2, Medical Faculty Mannheim, University of Heidelberg) approved this study. All participants gave written informed consent before study entry.

### Study design/Inclusion and exclusion criteria

We included healthy elderly subjects aged >55 years with no or only minimal disability in their instrumental activities of daily living (IADL). Exclusion criteria were a history of stroke or other neurological or psychiatric diseases, head and neck surgery, hearing loss, impaired vision or physical disabilities, left-handedness and contraindications to perform MRI. Furthermore duplex ultrasound examinations of the carotid arteries and the arteries of the circle of Willis were performed to exclude arterial stenosis. Twenty-five subjects participated in the study. The participants were split according to the extent of ARWMC in two groups with a low and a high lesion load (see data analysis). The data sets of nine subjects had to be discarded due to excessive motion during the fMRI experiment (see data analysis). Two further subjects in the low lesion load group were excluded to adjust both groups with respect to sample size and age, resulting in groups with seven participants each. For subject characteristics see *Table 1*.

**Table 1:** Subject characteristics.

	Low lesion load	High lesion load	P value
Number of subjects (N)	7	7	
Age, years (median, range)	66 (55-72)	68 (56-79)	0.427
Sex, male (N)	3	5	0.127
MMSE (median, range)	30 (28-30)	30 (28-30)	0.701
Education, high degree (N)	3	2	0.403
IADL (median, range)	8 (7-8)	8 (8-8)	0.655
Hypertension (N)	3	6	0.022 *
Diabetes mellitus (N)	2	3	0.403
Hyperlipidemia (N)	2	3	0.403
Smoking (N)	0	0	1.000

### Stimuli

Before the experiment itself, participants were instructed and practised the tasks. Three cognitive tasks were presented blockwise during fMRI. 1.) We used an alertness task to assess basic attentional function. During this task, the digit “2” was presented visually for 1 s in pseudo-randomized intervals between 1.2 s and 2.8 s. During a 36 s period 18 stimuli were presented to which subjects were asked to respond as fast as possible by pressing the button on a response device in the right hand. 2.) To assess attention we used a 0-back task. In this task, random numbers from 1–9 were presented. Subjects had to respond by pressing the button, when the number “2” was displayed. In one task block, 18 numbers appeared in intervals of 2 s (1 s on, 1 s off). 3.) Finally, a 2-back working memory task was used, during which the numbers were presented in the same way as in the 0-back task. A response was requested if a currently shown number was identical to the second last one.

### Data acquisition

During the fMRI measurement, the stimulus was presented as a movie with the use of the “Integrated Functional Imaging System” (IFIS, Invivo, Orlando, USA) via an LCD screen attached to the MRI head coil. Subjects with visual impairment were provided with MR-compatible corrective lenses. Performance of subjects was monitored visually and via the IFIS response units. The MR scan was performed on a 1.5 T whole body scanner (Magnetom Sonata, Siemens Medical, Erlangen, Germany). A three-dimensional T1-weighted whole brain data set was acquired (MPRAGE; TR/TE/flip angle = 1.900 ms/3.9 ms/15°) with an isotropic resolution of 1×1×1 mm^3^ for anatomical reference. A FLAIR data set was acquired to identify individual lesion load (TR/TE/TI/turbo factor = 9000 ms/108 ms/2400 ms/25; voxel size 1×1×4 mm^3^). For the three BOLD fMRI scans, a T2*w EPI sequence was used (TR/TE/flip angle = 2000 ms/55 ms/90°) with an in-plane resolution of 4×4 mm^2^. Per volume, 20 slices (4 mm thick, 2 mm gap) parallel to the inferior borders of the corpus callosum were scanned in interleaved order. For each fMRI experiment an identical blocked design was used which consisted of five baseline blocks of 18 volumes (black screen with fixation cross, 1 s on, 1 s off) and four task blocks of 18 volumes. Each fMRI scan started with two dummy scans which were discarded automatically by the scanner to minimize non-equilibrium T1 effects, resulting in a total measurement time of 5 min 28 s. Anatomical data sets and all three fMRI runs were scanned in one session.

### Data analysis

#### Structural and functional MRI data were analysed by independent investigators

The degree of ARWMC severity on MRI was rated using a modified version of the visual scale of Fazekas [11], ranging from 0 to 3, that scores deep and subcortical white matter lesions in three categories of mild (1), moderate (2), and severe (3) ARWMC. The study participants were classified into 2 groups: subjects with no or mild ARWMC (0–1) and those with moderate to severe ARWMC (2–3).

The fMRI data sets were pre-processed using AFNI [12]. After slice timing correction, a rigid body six-parameter motion correction was performed for each of the fMRI runs. Spatial smoothing with a Gaussian filter (FWHM = 8 mm) was applied to the data, and global intensity normalization was carried out. The T1-weighted anatomical data sets were then realigned to the EPI volumes. Statistical maps were created for each subject and for each task by performing a multiple linear regression (MLR) analysis. The ideal function was modelled as a boxcar function convolved with the hemodynamic response function. In the MLR, the whole brain signal time course and motion parameters were treated as regressors of no interest. The resulting signal per cent change maps (activation versus baseline) were transformed to Talairach space [13] using the transformation parameters of the respective anatomical data set. For the second level analysis, only those subjects were included with less than 2 mm absolute motion in all three fMRI experiments. Thus, the signal change maps of seven subjects in the low lesion load group and of seven subjects in the high lesion load group underwent a three-factor analysis of variance (ANOVA). Group and task were treated as fixed factors and the subjects as the random factor (AxBxC(A)-ANOVA). The following statistical maps were created as we have described in a previous study [14]: first, a main effect map for each task pooled over all subjects; second, a contrast map between both groups for each task; and third, the different task conditions contrasted separately for each group. The resulting t-maps were converted to Gaussian Z-scores and thresholded at Z>3.3 and at a corrected cluster significance level of p<0.01. Based on the group/task contrast map, the average per cent signal change of specific brain areas was evaluated for both groups separately. Regions of interest (ROI) were located in areas that showed either a significant task effect for the low lesion load group alone or for both groups [14]. Separately for both groups, the mean regional per cent signal change for each task was calculated and t-tests were performed.

## Results

**Figure 1:**
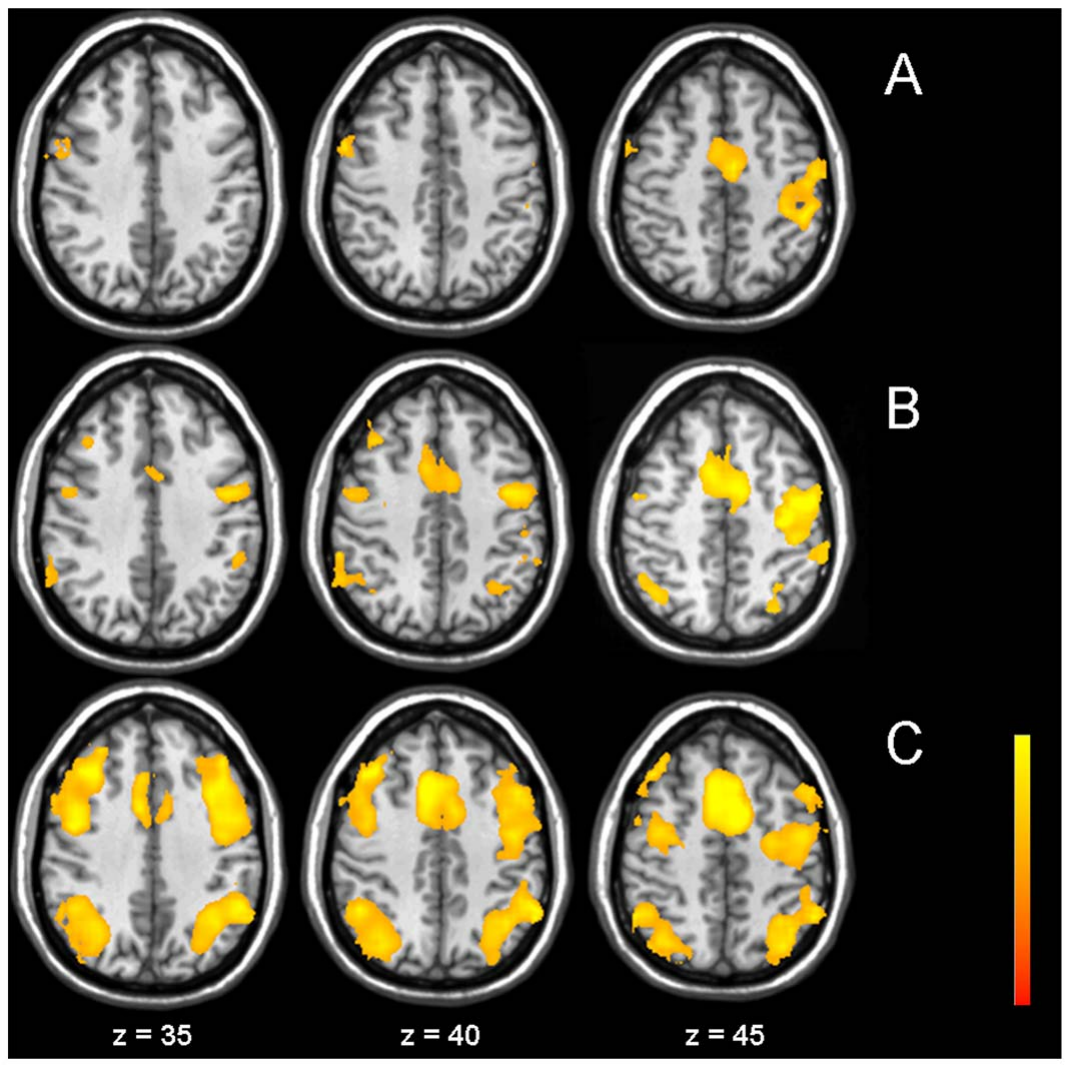
fMRI main effects. Group activation map over all subjects for the three different tasks alertness (A), 0-back (B), and 2-back (C) (Z>3.3, corrected p<0.01). Statistical maps are superimposed onto the MNI structural template. All MR Images shown here are in radiological order (image left is anatomical right).

For patient characteristics see *Table 1*. ARWMC grades were distributed as follows: Fazekas 0 in 1, Fazekas 1 in 6, Fazekas 2 in 3 and Fazekas 3 in 4 subjects, respectively. Data analysis of the study population (n = 14) showed recruitment of cortical areas parallel to increasing task difficulty (see *Figure 1* and *Table 2*). During all three tasks, the anterior insula (AI) bilaterally, as well as medial frontal and precentral areas were significantly activated. In the n-back tasks, additionally recruitment of bilateral posterior parietal areas was found.

**Table 2:** Areas of activated regions in the three different tasks (pooled over all subjects).

	Talairach-Tournoux Coordinates (mm)			max. Z-scores	p-values (corrected)
	x	y	z		
**Alertness – Main effect**					
Left medial frontal/left SMA	−5	−8	51	5.44	<0.01
Left precentral/postcentral	−46	−17	48	4.29	<0.01
Left superior temporal	−49	−41	28	4.01	<0.01
Right anterior insula	45	12	0	5.18	<0.01
Left anterior insula	−45	1	−2	4.06	<0.01
Right inferior/middle temporal	56	−63	−3	4.45	<0.01
Right cerebellum	19	−50	−22	4.09	<0.01
Left cerebellum	−33	−55	−26	4.73	<0.01
**0-back – Main effect**					
Left superior parietal	−32	−59	59	3.43	<0.01
Medial frontal/SMA	1	−8	58	5.00	<0.01
Right inferior/superior parietal	38	−62	46	3.18	<0.01
Left precentral	−33	−20	45	4.52	<0.01
Right middle/superior temporal	51	−41	9	5.26	<0.01
Right anterior insula	38	15	8	4.53	<0.01
Left anterior insula	−33	16	7	4.36	<0.01
Left cerebellum	−36	−51	−27	3.43	<0.01
Right precentral gyrus	38	−16	59	3.59	<0.01
**2-back – Main effect**					
Right middle/superior frontal/right precentral	28	−4	53	5.04	<0.01
Left middle/superior frontal/left precentral	−46	−10	49	4.2	<0.01
Medial frontal/anterior cingulate	−8	5	47	5.58	<0.01
Right inferior/superior parietal/right angular	45	−49	40	4.21	<0.01
Left inferior/superior parietal/left angular	−37	−57	38	4.43	<0.01
Left inferior frontal	−41	0	23	5.27	<0.01
Right inferior frontal	45	3	21	5.11	<0.01
Left anterior insula	−44	11	8	6.24	<0.01
Right anterior insula	35	17	7	5.82	<0.01
Right inferior temporal	49	−53	−15	3.55	<0.01
Left cerebellum	−33	−49	−32	5.05	<0.01
Right cerebellum	24	−59	−36	3.84	<0.01

**Figure 2:**
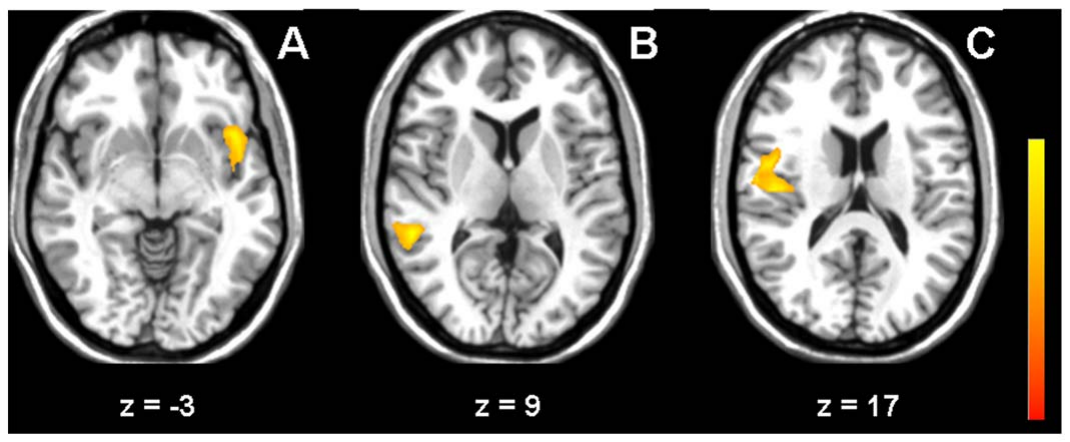
Effect of ARWMC lesion load. Difference in activation between the high lesion load group and the low lesion load group for the tasks alertness (A), 0-back (B) and 2-back (C) (Z>3.3, corrected p<0.01).

During all three tasks, the high lesion load group showed slightly higher activation compared to the low lesion group. In the alertness task, higher activation was found in the left anterior insula/inferior frontal cortex, in 0-back, differences were found in right middle temporal areas, and in 2-back in the right posterior insula/superior temporal gyrus (*Figure 2* and *Table 3*).

**Table 3:** Task main effect: differences between the low lesion load group and the high lesion load group.

	Talairach-Tournoux Coordinates (mm)			max. Z-scores	p-values (corrected)
	x	y	z		
**Alertness – Contrast high-low lesion load**					
Left insula/left inferior frontal	−42	13	−3	4.22	<0.01
**0-back – Contrast high-low lesion load**					
Right superior/middle temporal	51	−40	9	4.51	<0.05
**2-back – Contrast high-low lesion load**					
Right posterior insula/right superior temporal	45	−16	17	4.27	<0.01

**Figure 3:**
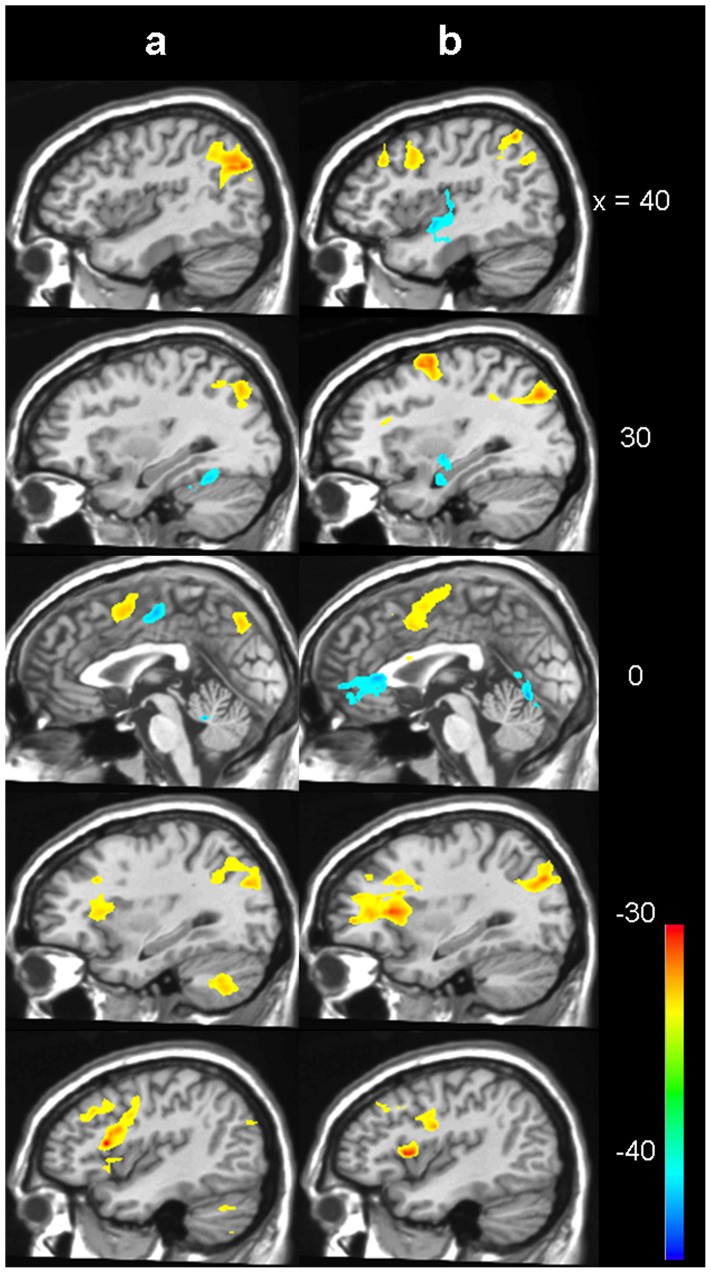
Increasing task difficulty in low lesion load. Activation change for the low lesion load group between 2-back and alertness (a) and between 2-back and 0-back (b). Blue to green means decreased activation for 2-back, yellow to red means increased activation (Z>3.3, corrected p<0.01).

**Figure 4:**
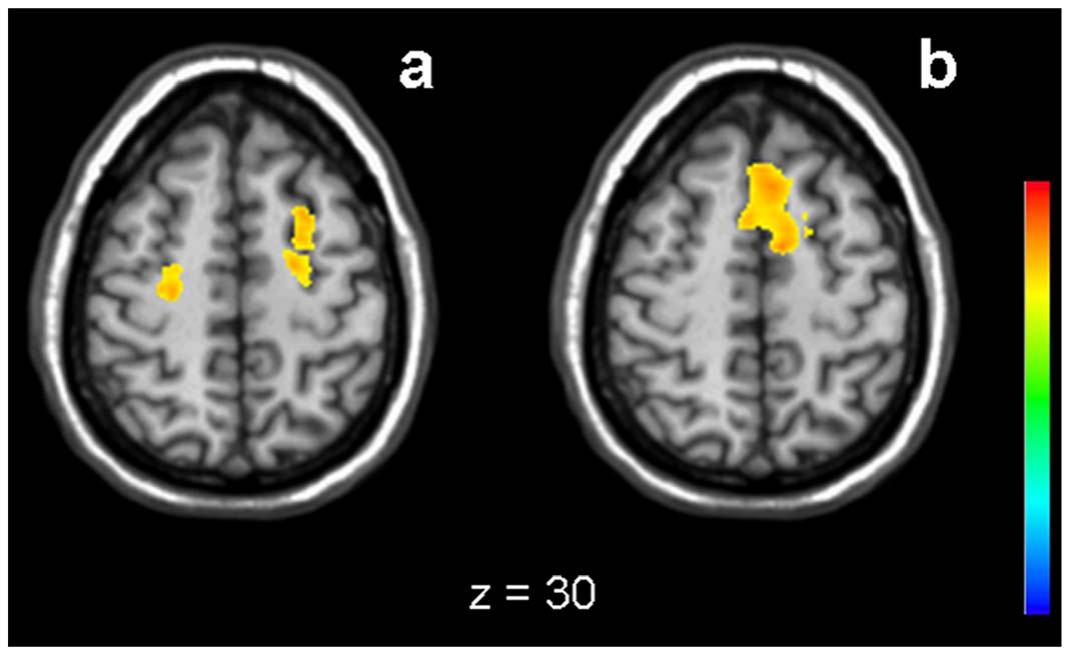
Increasing task difficulty in high lesion load. Activation change for the high lesion load group between 2-back and alertness (a) and between 2-back and 0-back (b). Yellow to red: increased activation (Z>3.3, corrected p<0.01).

There were significantly different characteristics of both groups with regard to the increase of activation parallel to task difficulty. In the low lesion load group, a significant and widely distributed change of activation in both 2-back versus 0-back, and 2-back versus alertness was observed (*Figure 3* and *Table 4*), including signal increase in medial frontal, bilateral parietal and dorsolateral frontal areas and decrease in the posterior insula and in cingulate and hippocampal areas. In contrast, in the high lesion load group only sparse increase of signal was found in medial frontal parts for 2-back versus 0-back and in bilateral middle frontal areas for 2-back versus alertness (*Figure 4* and *Table 5*).

**Table 4:** Changes between task classes in the low lesion load group.

	Talairach-Tournoux Coordinates (mm)			max. Z-scores	p-values (corrected)
	x	y	z		
**Contrast 2-back – alertness**					
Left medial frontal/left (pre-) SMA	−4	5	47	4.47	<0.01
Left precentral/left middle frontal	−41	25	35	4.23	<0.01
Right angular/right precuneus/right middle temporal	39	−72	31	4.42	<0.01
Left superior occipital/left angular/left precuneus/left middle temporal	−32	−75	27	3.88	<0.01
Left anterior insula/left inferior frontal	−41	16	14	5.35	<0.01
Left cerebellum	−42	−63	−27	3.63	<0.01
**Contrast alertness – 2back**					
Paracentral lobule/left medial frontal/left cingulate	14	−12	50	4.77	<0.01
Right insula/right postcentral	44	−15	11	4.77	<0.01
Right cerebellum	26	−47	−22	4.52	<0.01
**Contrast 2-back – 0-back**					
Cingulate/medial frontal	−5	2	48	4.29	<0.01
Right inferior parietal	43	−50	42	2.89	<0.01
Right middle frontal/right inferior frontal	42	9	38	3.81	<0.01
Right precuneus/right inferior parietal	29	−72	36	4.13	<0.01
Left precentral/left middle frontal	−32	18	34	3.12	<0.01
Left precuneus/left cuneus	−27	−76	30	4.77	<0.01
Left precentral/left inferior frontal	−37	−5	25	4.70	<0.01
Left precentral/left insula/left inferior frontal	−42	11	8	5.08	<0.01
**Contrast 0-back – 2back**					
Left posterior cingulate/left lingual	−9	−55	1	3.96	<0.01
Left anterior cingulate	−5	39	−3	3.93	<0.01
Left posterior insula	−39	−16	1	4.16	<0.05
Right insula	41	−6	−6	4.34	<0.05
Right hippocampus/right parahippocampal	20	−14	−10	2.86	<0.05
Left hippocampus/left parahippocampal	−32	−13	−12	3.83	<0.05
**Contrast 0-back – alertness**					
–	–	–	–	–	
**Contrast alertness – 0-back**					
–	–	–	–	–	

**Table 5:** Changes between task classes in the high lesion load group.

	Talairach-Tournoux Coordinates (mm)			max. Z-scores	p-values (corrected)
	x	y	z		
**Contrast 2-back – alertness**					
Left middle frontal	−15	−7	57	4.21	<0.05
Right middle frontal	19	−20	56	3.96	<0.05
**Contrast alertness – 2back**					
–	–	–	–	–	
**Contrast 2-back – 0-back**					
Left medial frontal	−8	−3	48	4	<0.01
**Contrast 0-back – 2back**					
–	–	–	–	–	
**Contrast 0-back – alertness**					
–	–	–	–	–	
**Contrast alertness – 0-back**					
–	–	–	–	–	

**Figure 5:**
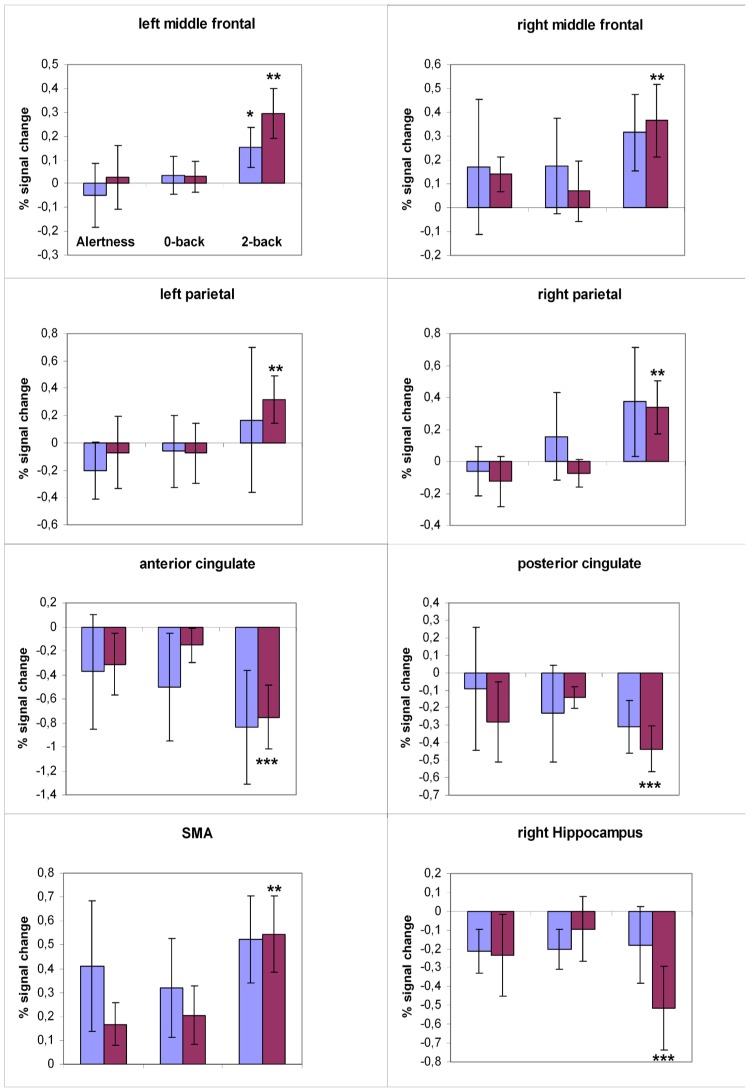
Quantification of task-dependent signal response. Regional mean percent signal change for different cortex areas defined by significant clusters in the task effect maps. The low lesion load group is shown in red bars, the high lesion load group in blue bars. The standard error is indicated by error bars. * significantly different to the alertness task (p<0.01); ** significantly different to the 0-back task (p<0.01); *** significantly different to both the alertness and 0-back tasks (p<0.01).

The ROI analysis of the signal change maps further characterized these effects (*Figure 5*). In the middle frontal cortex bilaterally, the supplemental motor area, and the right parietal cortex, the low lesion load group showed higher signal increase for the 2-back task compared to both the 0-back and the alertness task. In this group we also found a significant signal decrease between the 2-back and the 0-back task in the anterior and posterior cingulate gyrus and in the right hippocampal gyrus. Subjects in the high lesion load group showed higher activation even in the less demanding tasks in the right middle frontal cortex, in the supplemental motor area, and in the right parietal cortex. Accordingly, they demonstrated a decreased signal for the alertness and 0-back task in both anterior and posterior cingulated gyrus. Hence, the regional signal contrasts between the different tasks remained non-significant in this group of subjects.

## Discussion

While numerous studies have attributed a decrease in grey matter volume to cognitive decline and especially to working memory dysfunction, far less have addressed the effect of ARWMC on functional brain changes during working memory tasks. In this study we attempted to identify subclinical changes in brain activation in cognitive processing in individuals with different stages of asymptomatic ARWMC. We used a stimulation paradigm that provided very stable activation patterns. Although we had to discard nine datasets due to large motion artefacts in our cohort of elderly individuals, the paradigm itself was simple to perform and provided increasing degrees of complexity while using the same stimulation set-up ensuring robustness and the opportunity to relate the findings of each part of the experiment to the other.

Task specific activation patterns showed only subtle differences between the subjects with low or high ARWMC lesion load. In all three tasks, activation was found in the motor network including the contralateral primary motor cortex and the supplemental motor area, as expected for the motor response. Additionally, activation was found in the AI bilaterally in all three tasks. The AI is considered to integrate the sensory signals associated with voluntary movements [15]. A recent study also suggested that the bilateral AI may exert executive control by selectively biasing processing in favour of task-relevant information in an fMRI study investigating the neural correlates of perceptual load induced attentional selection [16]. Roski et al. analysed age-dependent differences and observed both selective increases and decreases in resting-state functional connectivity with age in regions associated with both attention and sensorimotor systems (rostral supplementary motor area and bilateral anterior insula) [17]. In both short-term memory tasks, a typical pattern of activation was found including bilateral dorsolateral prefrontal (BA9/46), inferior frontal (BA6/44) and parietal (BA7/40) areas. This pattern became more prominent with higher memory load. This result parallels findings in other studies using N-back paradigms [14], [18]–[20]. The inferior parietal cortex (BA40) is considered a locus of the storage component of working memory, whereas the inferior frontal cortex is involved in the rehearsal process [21], [22].

We found distinct changes of brain activity with increasing difficulty of the tasks. Subjects with a low lesion load showed a variety of activation differences between the three tasks, namely a signal increase parallel to task difficulty, but also recruitment of additional cortical areas and signal decrease in other areas such as the cingulum, the right insula, and the hippocampus. In contrast, only sparse task-dependent activation changes could be observed in the group with high lesion load. It might seem contradictory that we found distinct task-dependent activity changes for both groups, but almost no inter-group task effect differences. In several regions including the anterior cingulum, the right middle frontal gyrus, and supplemental motor areas, the activation level in the high lesion load group was already higher during the simplest task (alertness) than in subjects with low lesion load. In other regions such as the middle frontal areas in both hemispheres and right hippocampus, the maximal BOLD amplitude for the 2-back task was lower in the high lesion load group. Due to the lower BOLD signal range, the inter-task differences in subjects with high lesion load remained non-significant. Additionally, higher inter-individual variances were observed in the high lesion load group, particularly in bilateral parietal and cingulate areas, which also led to a reduced statistical significance. The higher inter-individual variances could also be an indication of different functional compensatory mechanisms with respect to the variable impairments of a higher lesion load.

All these findings may be interpreted as normal adaptation processes to task complexity in low ARWMC lesion load, while it could be postulated that those subjects with a high lesions load may already have compensatory activation at the level of the simplest task. In a very recent fMRI study of motor performance examining a cohort quite similar to ours, Linortner et al. observed an increased recruitment of supplemental motor areas in individuals with advanced ARWMC in a simple motor paradigm of ankle movement representing an element of gait. In line with our interpretations, they conclude that the alterations match the clinical phenomenology of SVE and speculate that a disruption of frontosubcortical networks might be its pathoanatomical correlate [23]. Nordahl and co-workers used ARWMC as a marker for white matter degeneration to demonstrate that increases in both global and regional dorsal prefrontal cortex ARWMC volume were associated with decreases in prefrontal cortex activity and decreased activity in the posterior parietal and anterior cingulate cortex during working memory performance [24]. Charlton et al. performed MRI and cognitive testing in 84 middle-aged and elderly adults at baseline and after two years and showed a correlation of diffusion tensor imaging (DTI) in white matter histograms with a change in working memory function [25]. A more recent study found significant increases in fMRI activation in the left dorsal and ventral lateral prefrontal cortices with increased working memory load and also with increased age that correlated with DTI derived fractional anisotropy (FA) in frontal brain regions [26]. A study addressing a similar hypothesis as ours, reported a reduction in the up-regulation of prefrontal and parietal regions in response to increasing working memory task demand along with a reduction in the down-regulation of default mode network regions with increasing cognitive load in the elderly compared to a younger group [27]. However the study did not report ARWMC. More pronounced brain activation might be interpreted as potential additional recruitment or reduction of inhibitory activity. Both have been interpreted as compensatory mechanisms preserving neurological function in the presence of structural tissue damage. An fMRI study examining the correlates of age-related reduction in working memory capacity, found that at higher working memory loads with worse performance older subjects had relatively reduced activity in prefrontal regions implicating a decline in performance past a threshold of physiological compensation [28].

Theoretically, different degrees of microangiopathy could lead to different BOLD responses. In functional magnetic resonance imaging (fMRI), neuronal activity is detected indirectly via the blood oxygenation level dependent (BOLD) effect [29]. Neuronal events induce an increase in local blood flow, but can also alter the BOLD signal by influencing several other factors like cerebral blood volume and cerebral blood oxygen consumption. This neurovascular coupling could be altered by microvascular disease thus affecting the BOLD signal. The anatomical scans prior to our fMRI experiments showed that none of the subjects had asymptomatic incidental pathology (e.g. lacunar stroke, cortical lesions). To exclude subtle influences of microangiopathy to the fMRI results, we focussed on group-by-task interactions and not on main effects in BOLD response between both groups [30]. Additionally, the ROI analysis demonstrated that the maximum amplitude of signal change was similar in both groups, which may indicate a comparable response range to stimulation. This would argue against differences induced by vascular pathology and microangiopathy. However, our study has two main limitations: first, the small sample size for an fMRI cohort study [31]. To preserve a high standard of our data we had to exclude a number of subjects due to intolerable movement artefacts. It has to be taken into account that our study cohort consisted of healthy but aged individuals. In contrast to young volunteers in many cognitive fMRI studies, our participants had no previous experience with being examined in an MR scanner or even with using computers as necessary for the cognitive tasks. Second, the performance data could not be used as regressors because a technical transmission error lead to incomplete data recording. However, the paradigms were designed and reviewed to be feasible for our cohort. All subjects were able to solve the paradigms in the training runs, and during the fMRI experiments their responses were continuously monitored.

The results of this study indicate that clinically silent ARWMC may affect cognitive processing and lead to compensatory activity in cognitive tasks. This can be interpreted as a reduction of functional reserve and may pose a risk for cognitive decline in these patients. However, these hypotheses need further substantiation in a consecutive study with larger patient numbers.
